# Supplementary material for: Membrane depolarization kills dormant Bacillus subtilis cells by generating a lethal dose of ROS
Source: Nat Commun. 2024 Aug 11;15:6877. doi: 10.1038/s41467-024-51347-0 (PMC11317493; doi:10.1038/s41467-024-51347-0)
Supplement: Supplementary file 1 — Supplementary Information [file 41467_2024_51347_MOESM1_ESM.pdf]

Supplementary Information

Membrane depolarization kills dormant *Bacillus subtilis* cells by generating a lethal dose of ROS

Declan A. Gray, Biwen Wang, Margareth Sidarta, Fabián A Cornejo, Julian Wijnheijmer, Rupa Rani, Pamela Gamba, Kürşad Turgay, Michaela Wenzel, Henrik Strahl, Leendert W. Hamoen

Table of content

- Table S1. Strains used in this study
- Table S2. Plasmids used in this study
- Table S3. Primers used in this study
- Table S4. Minimal inhibitory concentrations (MICs) of antibiotic compounds used in this study against *B. subtilis* PG344
- Table S5. Fractional inhibitory concentration (FIC) and FIC index (FICI) of antibiotic compounds.
- Fig. S1. Membrane depolarization of *B. subtilis* cells in the exponential and stationary phase
- Fig. S2. pH of the growth media of different strains
- Fig. S3. Effect of cell density on the efficacy of valinomycin
- Fig. S4. Valinomycin killing of stationary phase autolysin and prophage mutants
- Fig. S5. Growth and stationary phase cell densities of mutants
- Fig. S6. ROS production measured by Oxyburst Green and Mitosox Red
- Fig. S7. Presence of tiron increases viability of a Δ*sodA* mutant
- Fig. S8. Paraquat does not affect the membrane potential
- Fig. S9. Post-stress recovery on plates with ROS scavengers
- Fig. S10. Effect of menaquinone depletion on valinomycin-induced killing
- Fig. S11. Δ*qcrA* deletion mitigates killing of a Δ*qcrBC* mutant
- Fig. S12. ROS production in the Δ*qcrA* mutant measured by Oxyburst Green and Mitosox Red
- Fig. S13. Large field fluorescence images of stationary phase cells expression cytochrome bc1 and cytochrome-c oxidase subunits and cytochrome c550 fused to GFP
- Fig. S14. Large field fluorescence image of exponential phase cells expression cytochrome bc1 and cytochrome-c oxidase subunits and cytochrome c550 fused to GFP
- Fig. S15. Killing of stationary phase *S. aureus* cells by valinomycin
- Supplementary References

Table S1. Strains used in this study

Strain	Genotype	Source or reference
2682	*recA::tet*	Lab strain
1A1299	*Δskin, Δprophage1, Δprophage3, ΔSPbeta, ΔPBSX, pks::Cm, ICEBs1(0)*	BGSC
BKE01830	*ndhF::erm*	BGSC
BKE08820	*katA::erm*	BGSC
BKE09300	*glpD::erm*	BGSC
BKE14590	*pdhB::erm*	BGSC
BKE14890	*ctaC::erm*	BGSC
BKE14900	*ctaD::erm*	BGSC
BKE14910	*ctaE::erm*	BGSC
BKE14920	*ctaF::erm*	BGSC
BKE17930	*ccdA::erm*	BGSC
BKE19330	*sodF::erm*	BGSC
BKE19400	*sodC::erm*	BGSC
BKE22540	*qcrC::erm*	BGSC
BKE22550	*qcrB::erm*	BGSC
BKE22560	*qcrA::erm*	BGSC
BKE23150	*resA::erm*	BGSC
BKE25020	*sodA::erm*	BGSC
BKE25190	*cccA::erm*	BGSC
BKE28450	*sdhC::erm*	BGSC
BKE35270	*cccB::erm*	BGSC
BKE38760	*cydA::erm*	BGSC
BSB1	*trp+*	Lab strain
BWB03	*spoIIE::spec, cccA::erm*	This work
BWB04	*spoIIE::spec, cccB::erm*	This work
BWB76	*spoIIE::erm, amyE::Pxyl-gfp-qcrA-spec*	This work
BWB77	*spoIIE::erm, amyE::Pxyl-gfp-qcrB-spec*	This work
BWB78	*spoIIE::erm, amyE::Pxyl-gfp-qcrC-spec*	This work
BWB79	*spoIIE::erm, amyE::Pxyl-gfp-cccA-spec*	This work
BWB80	*spoIIE::erm, amyE::Pxyl-gfp-ctaC-spec*	This work
BWB81	*spoIIE::erm, amyE::Pxyl-gfp- ctaD-spec*	This work
BWB92	*spoIIE::erm, qcrBC::kan*	This work
BWB93	*spoIIE::erm, qcrABC::kan*	This work
BWB96	*ΔspoIIE, recA::erm, qcrA::kan*	This work
DG001	*spoIIE::erm, divIVA-gfp*	[1]
DG003	*Δskin, Δprophage1, Δprophage3, ΔSPbeta, ΔPBSX, pks::Cm, ICEBs1(0), spoIIE::erm*	This work
DG010	*spoIIE::erm, recA::tet*	This work
DG022	*lytABC::neo, lytD::tet, lytE::chl, lytF::spc, spoIIE::erm*	This work
DG025	*spoIIE::erm, spx::kan*	This work
DG037	*spoIIE::erm, ΔsodA*	This work
DG038	*spoIIE::erm, ΔsodC*	This work
DG042	*spoIIE::erm, ΔsodF*	This work
DG051	*spoIIE::erm, ΔkatA*	This work
DG082	*spoIIE::spec, glpD::erm*	This work
DG102	*spoIIE::spec, pdhB::erm*	This work
DG103	*spoIIE::spec, sdhC::erm*	This work
DG106	*spoIIE::spec, qcrA::erm*	This work
DG110	*spoIIE::spec, qcrB::erm*	This work
DG111	*spoIIE::spec, cydA::erm*	This work
DG112	*spoIIE::spec, ctaE::erm*	This work
DG113	*spoIIE::spec, ccdA::erm*	This work
DG115	*spoIIE::spec, resA::erm*	This work
DG116	*spoIIE::spec, ctaC::erm*	This work
DG117	*spoIIE::spec, ctaF::erm*	This work
DG118	*spoIIE::spec, ctaD::erm*	This work
DG119	*spoIIE::spec, qcrC::erm*	This work
DG120	*ndh::erm, spoIIE::spec*	This work
DGYK1460	*spoIIE::erm, divIVA-gfp, ctaB::tn-kan*	This work
DGYK1461	*spoIIE::erm, divIVA-gfp::chl, yjlD(ndh)::tn-kan*	This work
DGYK1462	*spoIIE::erm, divIVA-gfp::chl, qoxB::tn-kan*	This work
PG344	*spoIIE::erm*	[1]
PG344_2	*spoIIE::spec*	[1]

SH1000	*Staphylococcus aureus wildtype Newman*	[2]
SH1000 ΔsodA	*S. aureus, sodA::erm*	[2]
SH1000 ΔsodAM	*S. aureus, sodA::erm, sodM::tet*	[2]
SH1000 ΔsodM	*S. aureus, sodM::tet*	[2]
YK1461	*yjlD(ndh)::tn-kan*	[3]
YK1462	*qoxB::tn-kan*	[3]
Δspx	*spx::kan*	Lab strain
FC67	*lacA::PxylA dcas9 erm*	This work
FC175	*lacA::PxylA dcas9 erm, amyE::Pveg sgRNA-menC chl*	This work
FC181	*lacA::PxylA dcas9 erm, amyE::Pveg sgRNA-rfp chl*	This work

Table S2. Plasmids used in this study

Plasmid	Genotype	Source or reference
pBW6	*amyE::(Pxyl-msfgfp-qcrC-spec)*	This work
pDR244	*cre, spec, amp*	BGSC
pErm::Spec	*erm::spec*	[4]
pHJS105	*amyE::(Pxyl-mSFgfpV206K spec)*	[5]
pLB2	*amyE::(Pxyl-msfgfp-qcrA-spec)*	This work
pLB4	*amyE::(Pxyl-msfgfp-ctaD-spec)*	This work
pLB6	*amyE::(Pxyl-msfgfp-ctaC-spec)*	This work
pLB8	*amyE::(Pxyl-msfgfp-qcrB-spec)*	This work
pJMP1	*lacA::PxylA dcas9 erm*	[6]
pJMP2	*amyE::Pveg sgRNA-rfp chl*	[6]
pJMP2-menC	*amyE::Pveg sgRNA-menC chl*	This work

Table S3. Primers used in this study.

Primer	*Sequence 5'—3'*	Gene
BW_100	TTAACAAAATTCTCCAGTCTTCA	Rv-amyE5'
BW_104	TCACATTTATATTGTGCAACACTTCACAAA	Fw-amyE3'
BW_105	TGACCCTCCAGAGCCACCTTTGTAGAGCTCATCCATGCCATGT	Fw-sfGFP3
BW_106	GGTGGCTCTGGAGGGTCAAAATCAAAGTTATCGATACTAATGAT	Fw-cccB
BW_107	TGGATCCGAAGTCTGGACATTTTttaCTTTTTTTCTGACAGCCATTT	Rv-cccB
BW_241	CTAAAACAATTCATCCAGTAAAATAT	Rv-Km
BW_242	TAAAGAGAATGTCATGACTACC	Fw-qcrAupstream
BW_245	TTTTGTGTGTCTGATGTATCAG	Rv-qcrBCdownstream
BW_247	TCCGGTGATATTCTCATTTTAGCCATCTCTTCTCCCCCCTCTAA	Rv-qcrAupstream
BW_299	TAAAATATTATATTTTACTGGATGAATTGTTTTAGCGTATTGTGAA TTGTCAAATGG	Fw-qcrBCdownstream
BW_303	ACATAGTATCGACGGAGCCGATTGTCACCCTTCCCCCTTAG	Rv-qcrAupstream2
BW_313	CTTTGCTTTTCCAAGATACAG	Rv-qcrAinternal
BW_314	CAATTTTTGAATTATACGCTCAC	Fw-qcrAinternal
BW_61	AAAATGTCCAGACTTCGGA	Fw-amyE5'
BW_64	AAAATGTCCAGACTTCGGATCCACatgCACCGGGGCAAAGGGAT	Fw-qcrC
BW_65	ATCAAGCTTATCGATACCGTCGACttaTTTTGCGGTTGTTTCAGAA AT	Rv-qcrC
BW_66	TGTCCAGACTTCGGATCCACATGGGCGGAAAACATGATAT	Fw-qcrA
BW_67	ATCAAGCTTATCGATACCGTCGACTCACCCTTCCCCCTTAGGCT	Rv-qcrA
BW_72	AAAATGTCCAGACTTCGGATCCACATGTTGAATGCGCTTACAGA	Fw-ctaD
BW_73	ATCAAGCTTATCGATACCGTCGACTTATGCCTTCACCCCTTTAT	Rv-ctaD
BW_86	ATGTCCAGACTTCGGATCCACATGGTAAAGCATTGGCGTC	Fw-ctaC
BW_87	GATCCGAAGTCTGGACATTTTCTACTTGCTTTCCGC	Rv-ctaC
BW_90	GGCTCCGGAAGTGGCTCTCTGAACAAAATTTATGAC	Fw-qcrB
BW_91	CGAAGTCTGGACATTTTTTTAAAGCGGTCCTGAAATCCC	Rv-qcrB
BW_92	ACTTCCGGAGCCGCTACCTTTGTAGAGCTCATCCAT	Rv-sfGFP2
BW_97	TGACCCTCCAGAGCCACCTTTGTAGAGCTCATCCATGC	Rv-sfGFP3
BW_98	GGTGGCTCTGGAGGGTCAAAATGGAACCCGCTTATTCC	Fw-cccA
BW_99	TGGATCCGAAGTCTGGACATTTTttaTTTAATTTTTGACACCCACT	Rv-cccA
EKP22	GTCGACGGTATCGATAAGCTTGAT	Fw-sfGFP
EKP30	GTGGATCCGAAGTCTGGACATTTT	Rv-sfGFP
MW245	AAAATGTCCAGACTTCGGATC	Fw-sfGFP2
Ters158	TCACATTTATATTGTGCAACACT	Rv-amyE3'
Ters350	CACCGCCGACATTCGCGTGGCTCCA	Rv-amyEDown
Ters351	GCATCAGGGCTGCGGCATCCGGA	Fw-amyEUp
ZT202	AATCGGCTCCGTCGATACTATG	Fw-Km
menC3sgRNA_F	attgaattcaGTTTTAGAGCTAGAAATAGC	pJMP2-sgRNA scaffold
menC3sgRNA_R	cacccttcagACATTTATTGTACAACACG	pJMP2-P_{veg}

Table S4. Minimal inhibitory concentrations (MICs) of antibiotic compounds used in this study against *B. subtilis* PG344.

Antibiotics	MIC	Funtional Target
Vancomycin	2 µg/ml	cell wall synthesis
Ciprofloxacin	0.2 µg/ml	DNA synthesis
Kanamycin	1 µg/ml	protein synthesis
Valinomycin	10 µM	membrane potential dissipation
Paraquat	2 mM	superoxide inducer

Table S5. Fractional inhibitory concentration (FIC) and FIC index (FICI) of antibiotic compounds.

Comp. 1	Comp. 2	MIC (mM)		FIC (mM)		FICI	STDEV	Note
		Comp. 1	Comp. 2	Comp.1	Comp. 2			
paraquat	thiourea	1.67	500	0.205	62.5	0.248	0.047	synergistic
valinomycin	thiourea	0.004	500	0.000505	23.85	0.174	0.018	synergistic
paraquat	tiron	1.67	125	>1.67	>125	>2	-	additive or antagonistic *
valinomycin	tiron	0.004	125	>0.004	>125	>2	-	additive or antagonistic*

*Here, it was not possible to determine whether effects are additive or antagonistic due to the limited solubility of tiron. Synergistic (FICI ≤0.5), additive (FICI >0.5 and <4) and antagonistic (FICI ≥4).

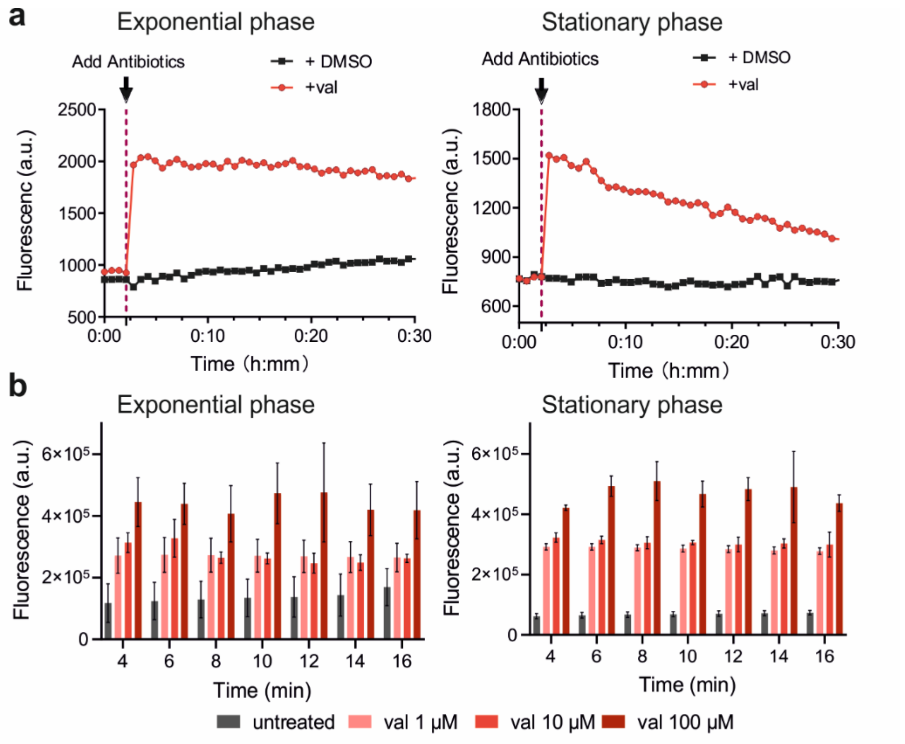

Fig. S1. Membrane depolarization of *B. subtilis* cells in the exponential and stationary phase.

a Membrane potential was measured using the membrane potential-sensitive fluorescent probe $DiSC_3(5)$ in microtiter plates. Cells from either exponentially growing or stationary phase cultures were incubated in microtiter plates, and when the baseline was stable, either 10 μM valinomycin (val) or 1 % DMSO was added to the cells. Arrows indicate the time point of valinomycin or DMSO addition. $DiSC_3(5)$ is taken up by cells driven by the membrane potential. The high concentration of intracellular $DiSC_3(5)$ causes fluorescence quenching, and depolarisation by valinomycin releases $DiSC_3(5)$ into the medium, resulting in an increase in fluorescence (red trajectories in the graphs). Three biological replicates were performed to ensure repeatability. **b** In the $DiSC_3(5)$ microtiter plate assay a ten-times lower valinomycin concentration was used than used in the experiments because 100 μM valinomycin begins to affect the fluorescence of $DiSC_3(5)$ in the microtiter plate assay. Therefore, we also tested an alternative microscopy approach whereby cells were first treated with 0, 1, 10 and 100 μM valinomycin for 60 min, and, after washing, incubated with $DiSC_3(5)$. When cells have a membrane potential they will take up $DiSC_3(5)$ from the buffer, lowering the fluorescence due to fluorescence quenching inside cells (black bars). The bar diagrams show that 10 and 100 μM valinomycin dissipate the membrane potential in comparable measures in exponentially growing cells, and that 100 μM works slightly better. Data reflect mean ± SD of three biological replicates.

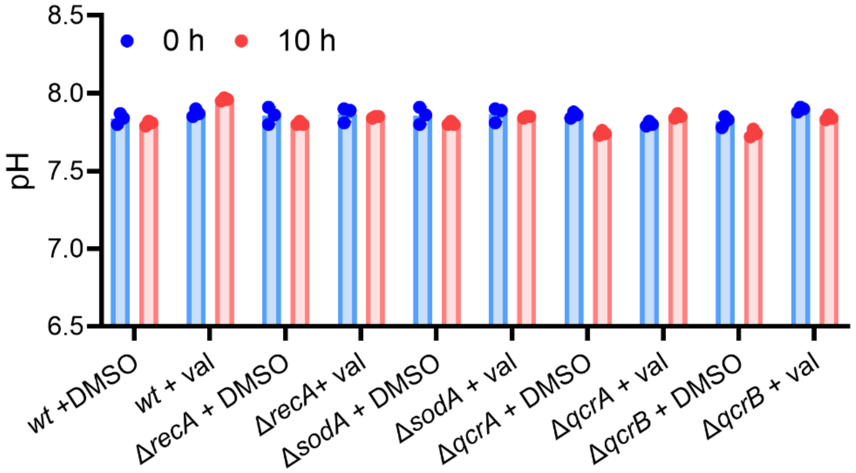

Fig. S2. pH of the growth media of different strains.

pH of different stationary phase cultures after 0 h and 10 h valinomycin treatment (val). The addition of 1 % DMSO was used as control. Data and mean of three biological replicates are shown.

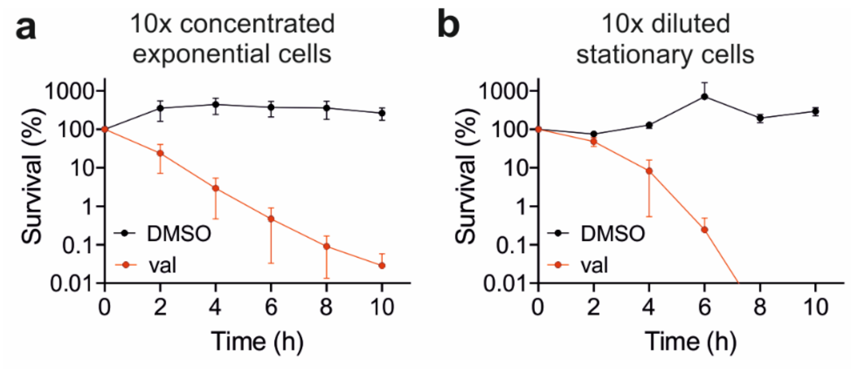

Fig. S3. Effect of cell density on the efficacy of valinomycin.

a Survival curve of 10x concentrated exponentially phase *B. subtilis* culture incubated with valinomycin. The OD_{600} of the cultures before concentration was approximately 0.5, and the addition of DMSO (1 %) was used as control. **b** Survival curve of 10x diluted stationary phase (dormant) *B. subtilis* culture incubated with valinomycin. The OD_{600} of the cultures before dilution was approximately 3.0. Exponential and stationary phase cultures were both resuspended in spent medium after centrifugation. The addition of 1 % DMSO was used as control. Mean and standard deviation of three biological replicates are shown.

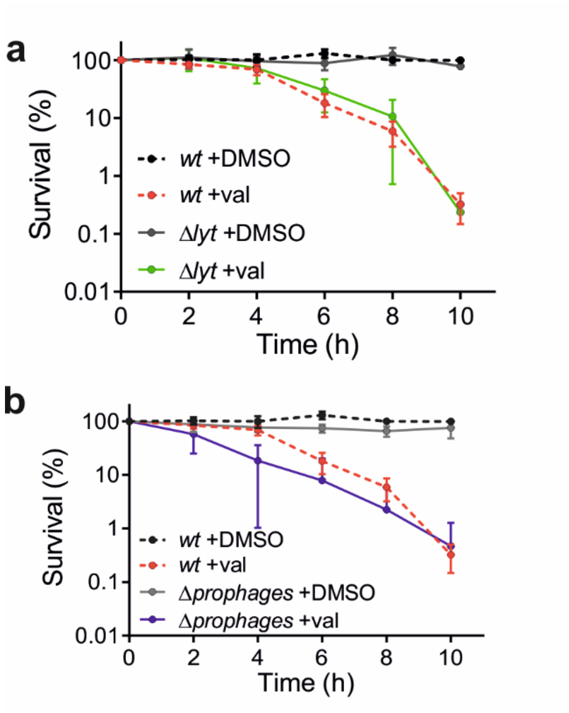

Fig. S4. Valinomycin killing of stationary phase autolysin and prophage mutants.

a Survival curves of a strain lacking the main autolysin encoding genes *lytA, -B, -C, -D, -E and -F* (Δ*lyt*). **b** Survival curves of a strain lacking all 6 prophages: *skin*, *ICEBs1*, *prophage1*, *prophage3*, SPβ, PBSX, and pks (Δ*prophages*). 100 µM valinomycin (val) and 1 % DMSO (w/w) were used. Mean and standard deviation of three biological replicates are shown.

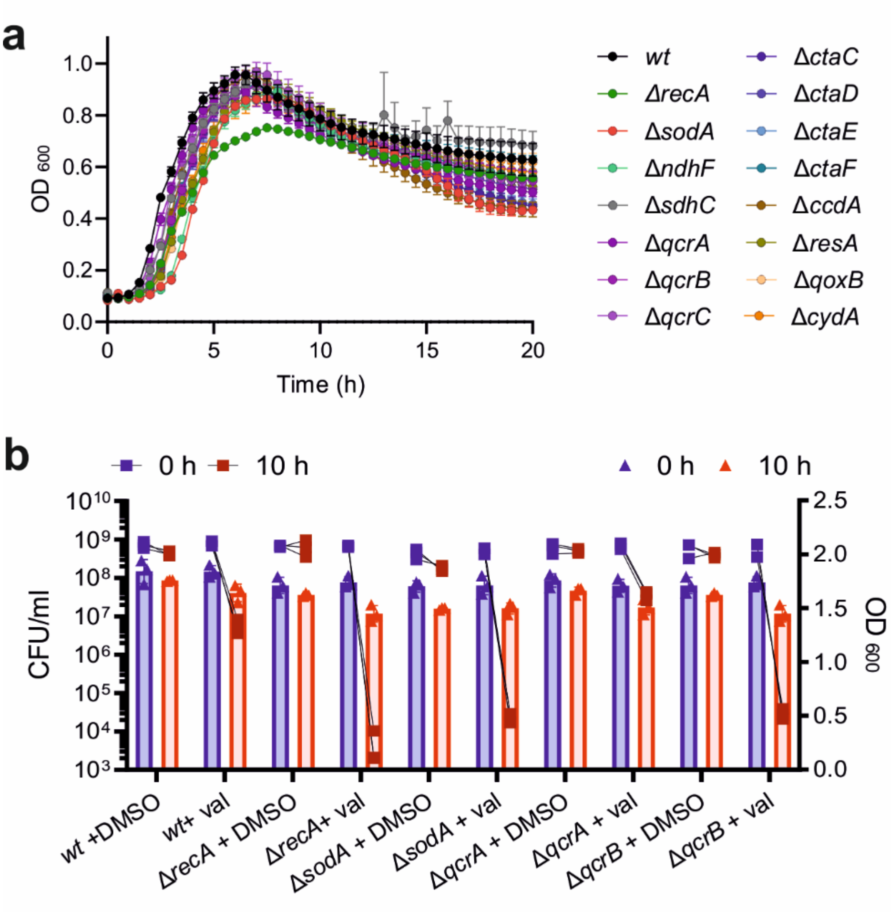

Fig. S5. Growth and stationary phase cell densities of mutants.

a Growth curves of mutants used in this study. The growth was measured in microtiter plates incubated at 37 °C for 20 h. The curves reflect mean and standard deviations of three biological replicates. Of note, the growth curves were measured in microtiter plates, which results in a stronger cell lysis during the stationary phase due to limited aeration. This is not the case in flaks used in the experiment. Data reflect mean ± SD of three biological replicates. **b** Optical densities (right Y-axis) of mutants incubated with valinomycin (val) for 0 h and 10 h, indicated with blue and red bars, respectively. The viable counts (left X-axis) are shown with squares and lines. 1 % DMSO was used as control. Data of three biological replicates are shown.

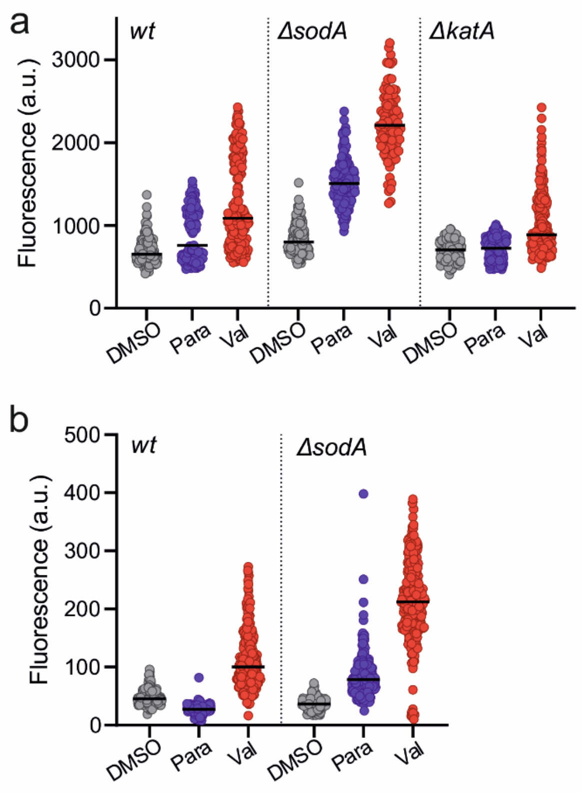

Fig. S6. ROS production measured by Oxyburst Green and Mitosox Red.

a ROS production measured with Oxyburst Green in stationary phase wild type (*wt*) Δ*sodA* and Δ*katA* cells after 2 h incubation with either 1 % DMSO, 1 mM paraquat (Para) or 100 µM valinomycin (Val). Fluorescence intensities of 100-200 cells were measured microscopically and plotted. Three biological replicates were performed to ensure repeatability. **b** Superoxide production measured with Mitosox Red in in stationary phase wild type (*wt*) and Δ*sodA* cells after 2 h incubation with either 1 % DMSO, 1 mM paraquat (Para), or 100 µM valinomycin (Val). Fluorescence intensities of 300 cells were measured microscopically and plotted. Three biological replicates were performed to ensure repeatability.

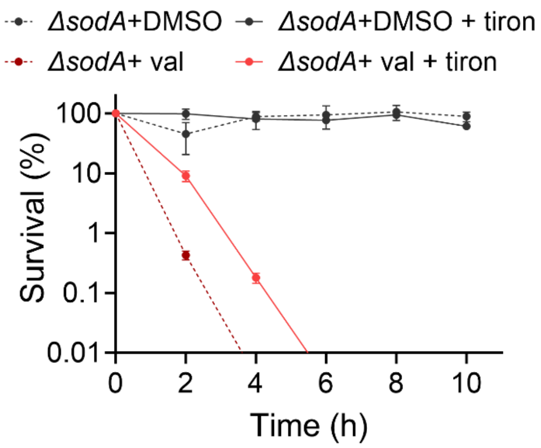

Fig. S7. Presence of tiron increases viability of a Δ*sodA* mutant.

Survival curves of a strain lacking the main superoxide dismutase SodA upon valinomycin (val) in the presence or absence of 10 mM tiron. Mean and standard deviation of three biological replicates are shown.

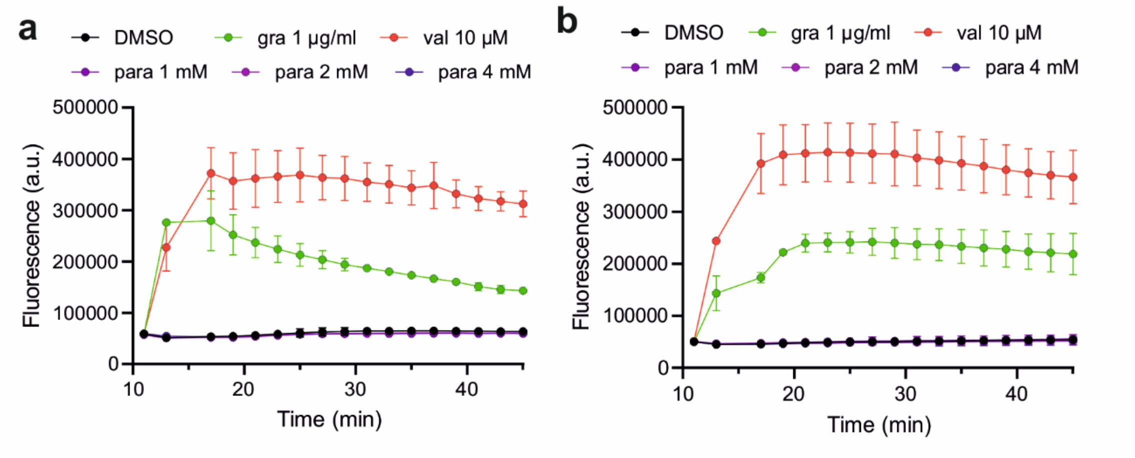

Fig. S8. Paraquat does not affect the membrane potential.

$DiSC_3(5)$ measurement of the membrane potential in the presence of different paraquat (para) concentrations. Cells from either exponentially phase (**a**) or stationary phase cultures (**b**) were tested. 1 µg/ml gramicidin (gra), 10 µm valinomycin (val) and 1 % DMSO were used as controls. Mean and standard deviation of three biological replicates are shown.

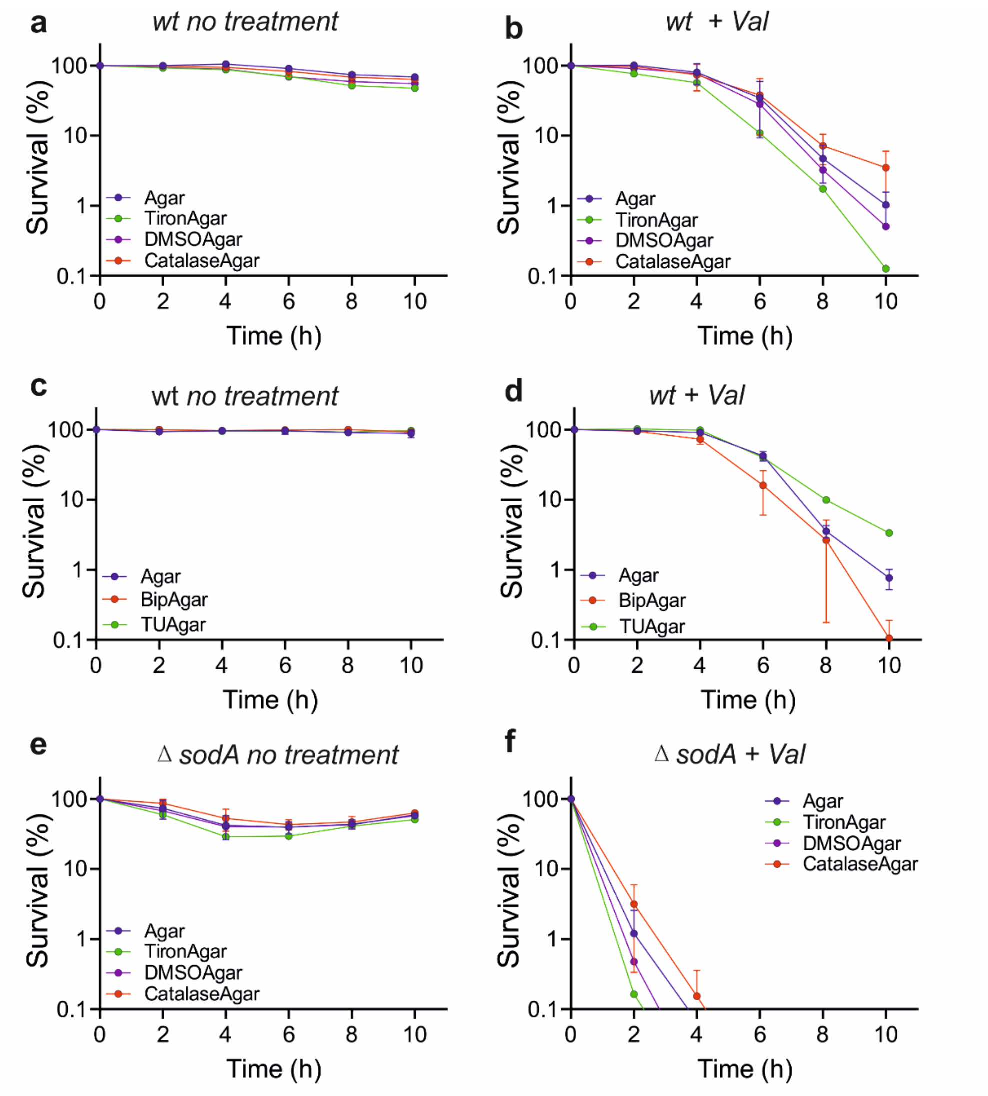

Fig. S9. Post-stress recovery on plates with ROS scavengers.

Survival curves of stationary phase *wt* (**a-d**) cells treated with either 100 µM valinomycin (val) (**b**, **d**) or with 1 % DMSO (no treatment) (**a**, **c**) for 10 h, and subsequent dilution on nutrient agar plates without supplements (Agar) or with either 10 mM tiron, 5 % DMSO, 200 U catalase (overlay) from *Aspergillus niger*, 3 µM bipyridyl (Bip) or 0.5 mM thiourea (TUAgar). (**e, f**) Survival curves of stationary phase Δ*sodA* cells treated with either 100 µM valinomycin (val, **f**) or with 1 % DMSO (no treatment, **e**) for 10 h, and subsequent dilution on nutrient agar plates without supplements (Agar) or with either 10 mM tiron, 5 % DMSO or 200 U catalase (overlay) from *Aspergillus niger*. Mean and standard deviation of three biological replicates are shown.

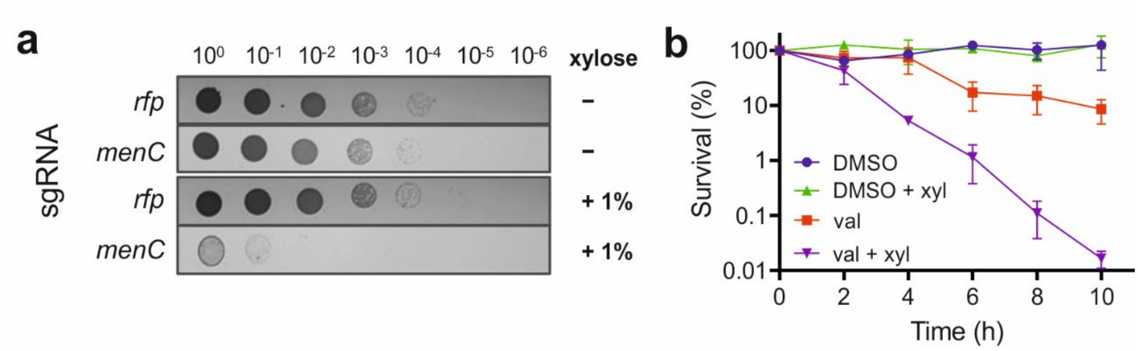

Fig. S10. Effect of menaquinone depletion on valinomycin-induced killing.

a CRISPRi knockdown of menaquinone synthesis (*menC*). Spot assay of *menC* interfered strain grown on $S7_{50}$ agar plates containing 1 % glycerol with or without xylose. The sgRNA was expressed under the constitutive promoter P_{veg}. Addition of 1 % xylose induced dCas9 expression, triggering transcriptional interference of *menC*. A strain with a non-targeting guide RNA directed against red fluorescent protein (*rfp*) was spotted as negative control. **b** Survival curves of normally expressing *menC* culture and a culture depleted for *menC* RNA due to the presence of 1 % xylose (xyl) in the absence or presence of valinomycin (val) or 1 % DMSO. Mean and standard deviation of three biological replicates are shown.

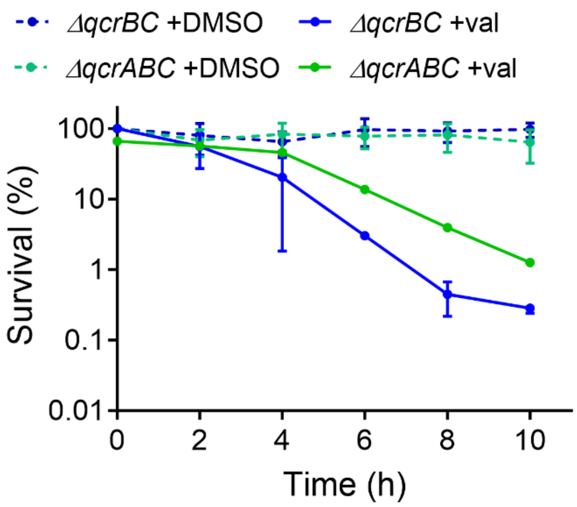

Fig. S11. Δ*qcrA* deletion mitigates killing of a Δ*qcrBC* mutant.

Survival curves of a Δ*qcrBC* and Δ*qcrABC* mutant treated with valinomycin (val) or 1 % DMSO. Mean and standard deviation of three biological replicates are shown.

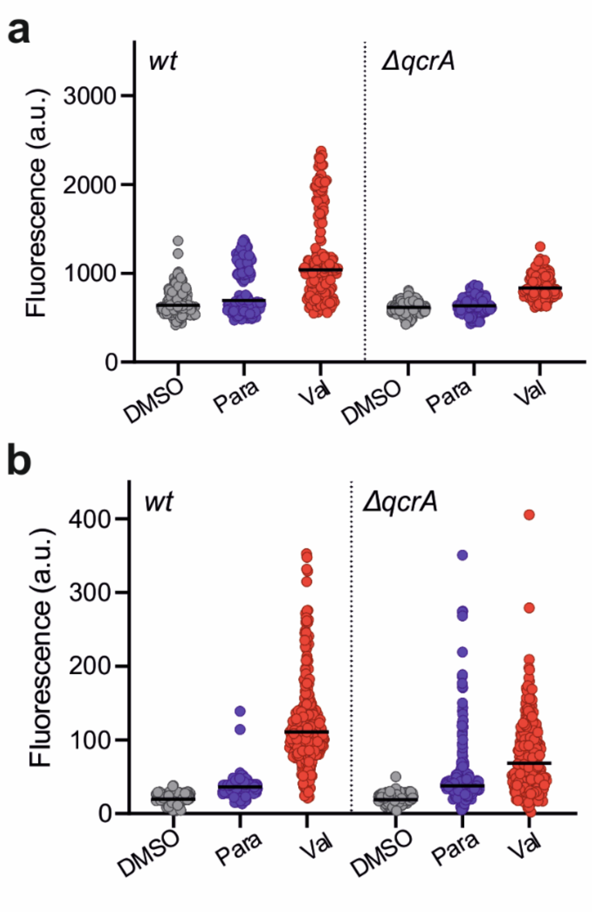

Fig. S12. ROS production in Δ*qcrA* mutant measured by Oxyburst Green and Mitosox Red.

a ROS production measured with Oxyburst Green in stationary phase wild type (*wt*) and Δ*qcr*A cells after 2 h incubation with either 1 % DMSO, 1 µM paraquat (Para) or 100 µM valinomycin (Val) for 2 h. Fluorescence intensities of 200 cells were measured microscopically and plotted. The wild type data is the same as previously shown in Fig. S6. Fluorescence intensities of 200 cells were measured microscopically and plotted. **b** Superoxide production measured with Mitosox Red in stationary phase wild type (*wt*) and Δ*qcrA* cells after 2 h incubation with either 1 % DMSO, 1 µM paraquat (Para), or 100 µM valinomycin (Val) for 2 h. Fluorescence intensities of 300 cells were measured microscopically and plotted. Three biological replicates were performed to ensure repeatability.

173

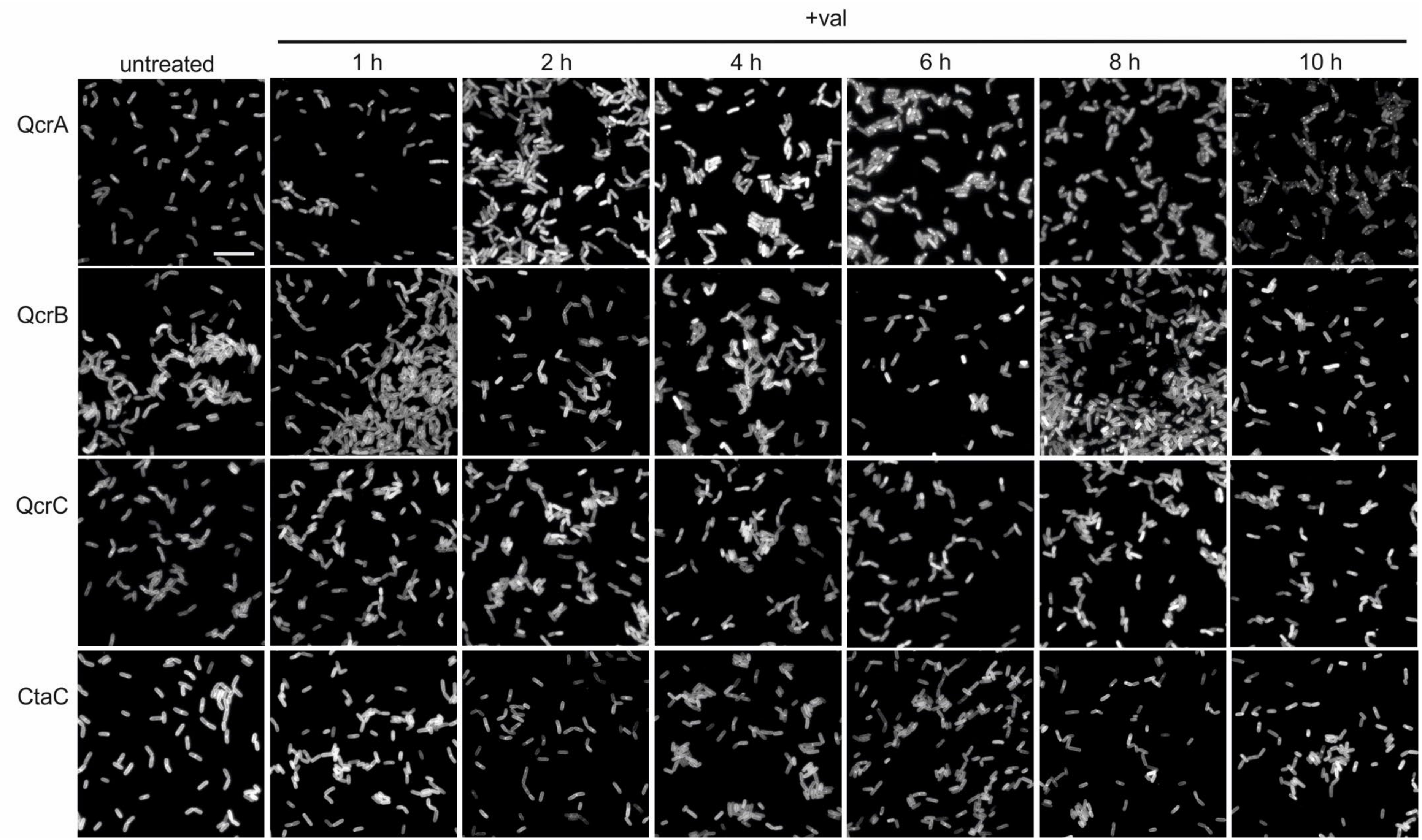

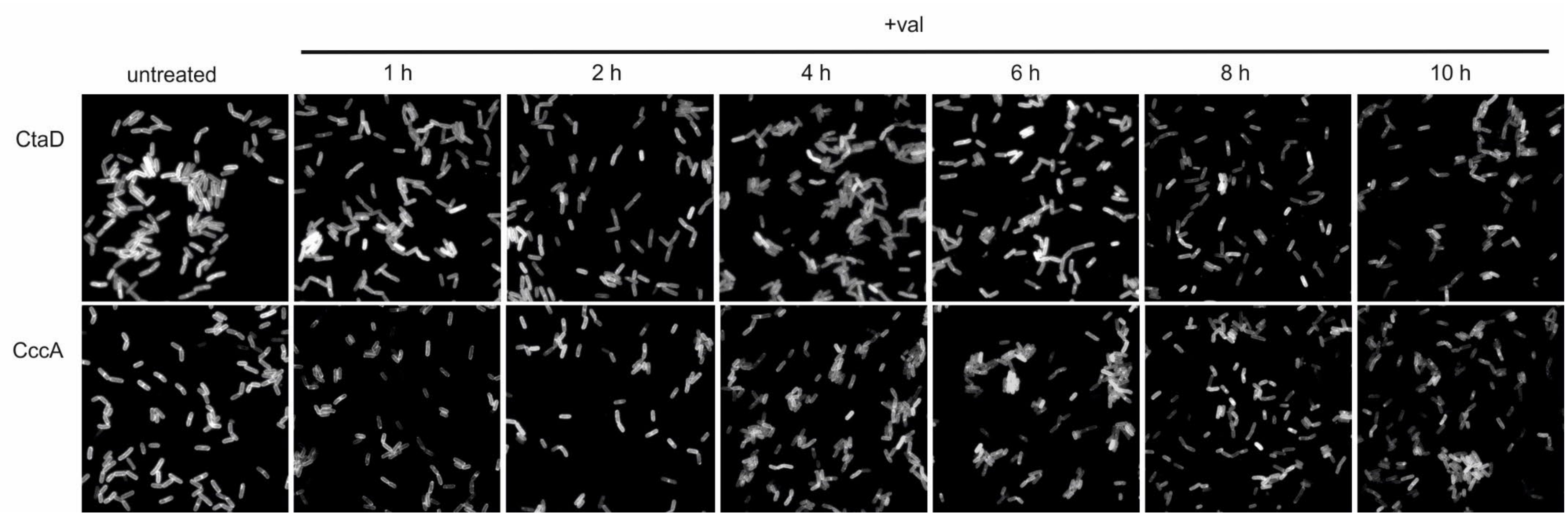

Fig. S13. Large field fluorescence images of stationary phase cells expressing cytochrome bc1 and cytochrome-c oxidase subunits and cytochrome c550 fused to GFP.

Cellular localization of GFP tagged cytochrome *bc*1 subunits QcrA (Rieske protein), QcrB, and QcrC, cytochrome *c* oxidase subunits CtaC and CtaD, and cytochrome *c*550 in stationary phase cells incubated with 100 μM valinomycin (+ val) for up to 10 h. Scale bar represents 10 μm. This figure is related to Fig. 6A in the main text. Three biological replicates were performed to ensure repeatability. Result of one biological replicate is shown.

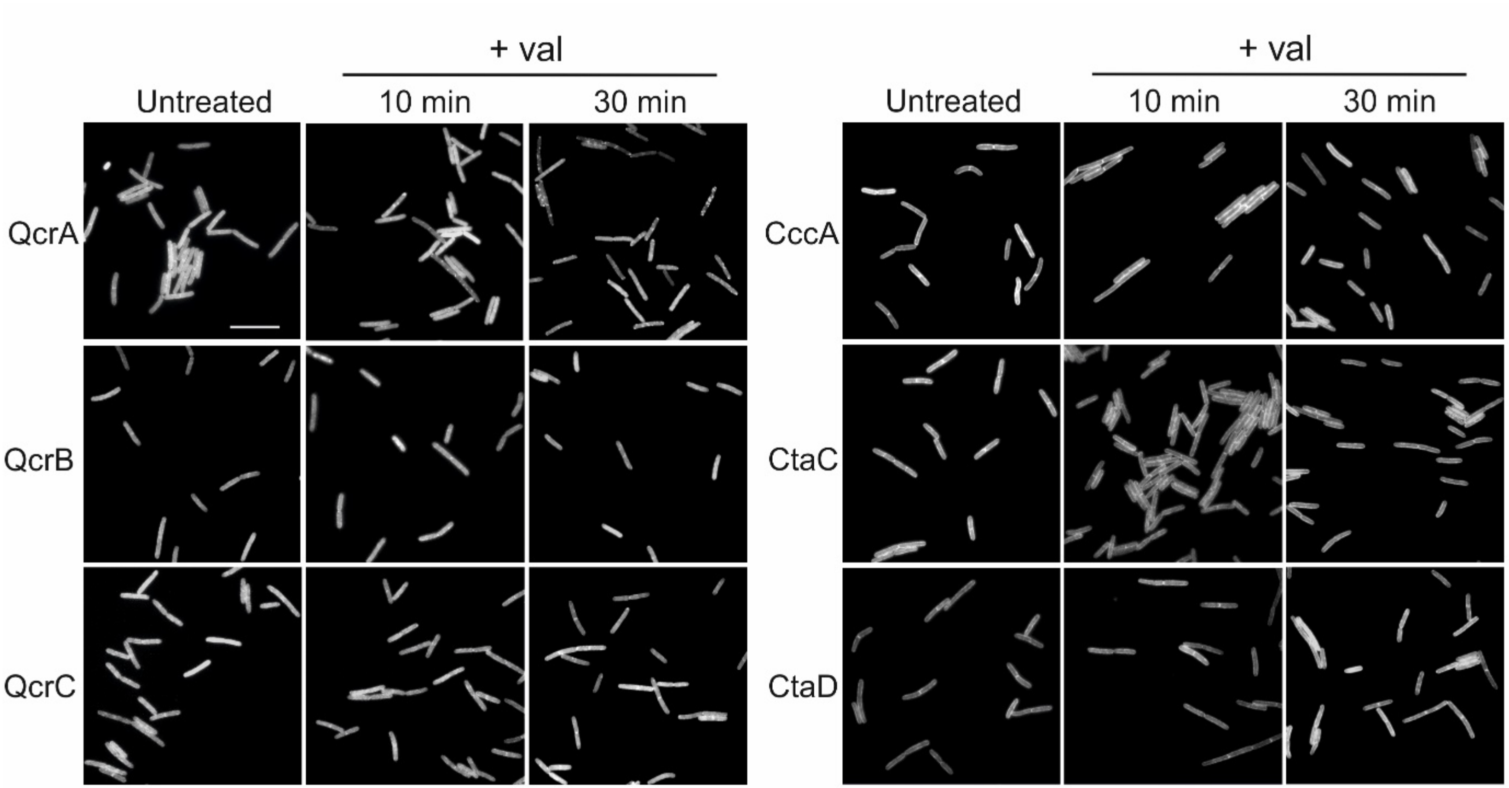

Fig. S14. Large field fluorescence image of exponential phase cells expressing cytochrome bc1 and cytochrome-c oxidase subunits and cytochrome c550 fused to GFP.

Cellular localization of GFP-tagged cytochrome *bc*1 subunits QcrA (Rieske protein), QcrB and QcrC, cytochrome *c* oxidase subunits CtaC and CtaD, and cytochrome *c*550 in exponentially growing cells incubated with 100 µM valinomycin (+ val) for up to 30 min. Scale bar represents 10 µm. This figure is related to Fig. 6B in the main text. Three biological replicates were performed to ensure repeatability. Result of one biological replicate is shown.

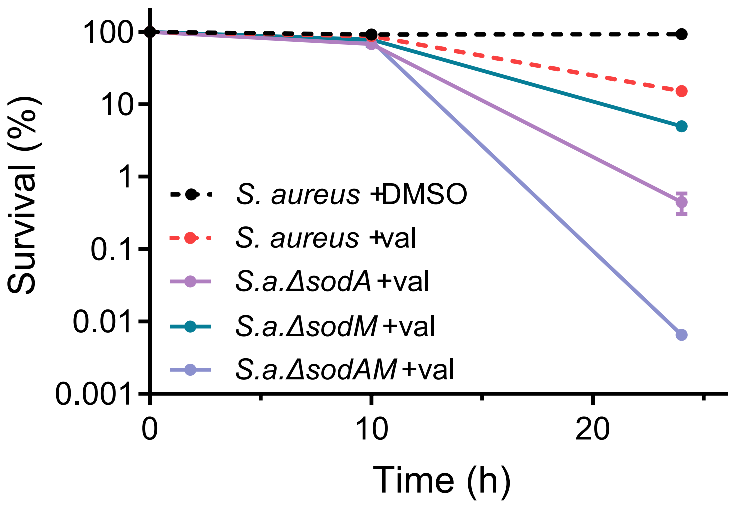

Fig. S15. Killing of stationary phase *S. aureus* cells by valinomycin.

Survival curves of stationary phase *S. aureus* wild type cells (*wt*) and the *S. aureus* superoxide dismutase mutants Δ*sodA*, Δ*sodM,* and double mutant Δ*sodAM* when incubated with 100 μM valinomycin (val). 1 % DMSO was used as negative control. The survival curves of the superoxide dismutase mutants with DMSO were similar to that of the *wt* strain and are not shown here. Mean and standard deviation of three biological replicates are shown.

Supplementary References

1. Gray, D. A. *et al.* Extreme slow growth as alternative strategy to survive deep starvation in bacteria. *Nat Commun* **10**, 890 (2019).
2. Tarrant, E. *et al.* Copper stress in *Staphylococcus aureus* leads to adaptive changes in central carbon metabolism. *Metallomics* **11**, 183–200 (2019).
3. Kawai, Y. *et al.* Cell Growth of Wall-Free L-Form Bacteria Is Limited by Oxidative Damage. *Current Biology* **25**, 1613–1618 (2015).
4. Steinmetz, M. & Richter, R. Plasmids designed to alter the antibiotic resistance expressed by insertion mutations in *Bacillus subtilis*, through in vivo recombination. *Gene* **142**, 79–83 (1994).
5. Gamba, P., Jonker, M. J. & Hamoen, L. W. A Novel Feedback Loop That Controls Bimodal Expression of Genetic Competence. *PLOS Genetics* **11**, e1005047 (2015).
6. Peters, J. M. *et al.* A Comprehensive, CRISPR-based Functional Analysis of Essential Genes in Bacteria. *Cell* **165**, 1493–1506 (2016).
